# GC-MS and E-Nose Analysis of Office Paper: Discriminating Paper Origin Using Multivariate Analysis

**DOI:** 10.3390/s26072049

**Published:** 2026-03-25

**Authors:** Marta I. S. Veríssimo, Elvira Gaspar, Maria Teresa S. R. Gomes

**Affiliations:** 1 Department of Chemistry, University of Aveiro, 3810-193 Aveiro, Portugal; mverissimo@ua.pt; 2 LAQV-REQUIMTE, Department of Chemistry, Faculty of Science and Technology, New University of Lisbon, Quinta da Torre, 2825-114 Caparica, Portugal; elvira.gaspar@fct.unl.pt; 3 Centre of Environmental and Marine Studies (CESAM), Department of Chemistry, University of Aveiro, 3810-193 Aveiro, Portugal

**Keywords:** GC-TOF-MS, electronic nose, piezoelectric quartz crystal, volatile organic compounds, office paper, wood fibers, paper wood, wood pulp

## Abstract

**Highlights:**

**What are the main findings?**

- An e-nose based on coated piezoelectric quartz crystals was used to detect VOCs emitted from office paper.
- The papers could be grouped based on the wood species used as the fiber source.

**What are the implications of the main findings?**

- VOCs emitted from paper can be used to identify the wood used in its manufacture, distinguish recycled paper, and infer its geographic origin based on differences in the manufacturing process.
- A new inexpensive and non-destructive analytical system is able to distinguish papers.

**Abstract:**

Volatile organic compounds (VOCs) emitted from hardwood papers are associated with cellulose fibers, paper fillers, and the manufacturing process used. Volatiles emitted from samples of office (printing and writing) papers from various brands and countries were analyzed by gas chromatography–mass spectrometry (GC-MS) and an electronic nose (e-nose) based on piezoelectric quartz crystals. Dodecanoic acid 1-methylethyl ester (isopropyl dodecanoate) and nonanal have shown to be the dominant compounds in most of the samples analyzed, regardless of the pulpwood used in paper manufacturing: Eucalyptus globulus, acacia, and birch. 3-Hydroxybutanone was detected only in Spanish papers, suggesting it as a potential marker. Additionally, the content in acetic acid enables the identification of recycled paper.

## 1. Introduction

Paper has been the material used for written communication and the dissemination of information. Paper (and paperboard) is also used for many other purposes, such as wrapping, packaging, toweling, and photography. Some papers, such as those used in banknotes, have strict manufacturing specifications, not only to ensure long durability but also to address security issues. Those include, for instance, watermarks, fluorescence fibers, and metallic fillets. Due to these specifications, paper traceability to its origin is assured.

Office (printing and writing) paper must also comply with certain specifications. Although they are not as strict as those for banknotes and security is not an issue, the final product must fulfil the functionality requirements. Office paper whiteness is an important parameter; thickness and grammage (paper weight per square meter, gsm) are also considered. Humidity must be controlled, and fillers and sizing agents must have been added and controlled during production so that resistance to wetting is ensured and ink running (bleeding, feathering) is prevented. Office paper has several utilizations, both at home and in the office; it is more commonly used for graphic printing. Therefore, the ink and printing methodology may vary, and handwriting should also be taken into account. Although a high degree of consistency in quality is required, variability may arise from wood fibers (as different plant species may be used in their production), from additives used in the paper-making process, and from other factors.

Among the trees used for office paper, *Eucalyptus globulus*, birch, aspen, oak, and acacia are mostly used [1]. They are all sources of hardwood fibers (with fiber lengths ranging from about 0.5 to 1.5 mm), which are shorter and thinner than softwood pulp fibers, and provide a smooth printing surface with high opacity [2]. Generally, hardwood fibers contain more cellulose and hemicellulose and less lignin than softwood, while softwood has a higher proportion of extractives, i.e., resin. Eucalyptus is the most common wood used for pulp production in Portugal. Acacia is a strong competitor in Asia due to propitious ecological conditions. Compared with *Eucalyptus globulus*, pulp yield from acacia can be higher and residual lignin content lower [3]. In addition, light scattering, which is important for writing and printing papers, was shown to be higher with acacia wood than with *E. globulus*, although eucalyptus fibers, at a given density, did show superior strength [3]. In Finland, Metsä produces birch-bleached hardwood kraft pulp (BHKP) [4]. It is also common to use blends of hardwood and softwood pulps to meet the desired paper properties.

Paper traceability is important in forensics, audits and certifications. Publishers need to ensure uniformity in re-editions and printing quality. Country of origin determines customs duties, and environmentally concerned citizens and ONGs need to prove that the wood does not come from protected forests and they are interested in assuring the inclusion of a minimum percentage of recycled paper.

Paper is ordinarily evaluated by its physical properties: density, air permeability, smoothness, stretch, stiffness, tensile index, opacity, and others [3]. Although office paper seems odourless, it can release volatile compounds originating from the wood itself and from the pulping, bleaching, and paper-making processes, including additives and fillers. Gas chromatography (GC) coupled with flame ionization detection (FID), electron capture detection (ECD), and mass spectrometry (MS) has been used to detect and identify volatile organic compounds (VOCs). These methods require expensive equipment, specialized operators, and a large database to compare the compounds. These techniques have already been used to study VOCs emitted from historical papers and old documents using SPME [5,6,7] or SKC passive (diffusive) samplers or “badges” [8]. However, as far as we know, there has been no research on new paper sheets that emit no perceptible VOCs and are not considered a cause for concern.

An electronic nose (e-nose) based on an array of piezoelectric quartz crystals was already used to analyze historical papers [9]. The method used was not only non-destructive but also inexpensive. A tailored electronic nose could help to identify distinctive VOC profiles from different papers by selecting an appropriate sensitive membrane for each sensor in the array.

According to Gardner and Bartlett [10], an electronic nose “is an instrument, which comprises an array of electronic chemical sensors with partial specificity and an appropriate pattern-recognition system, capable of recognising simple and complex odours”. Although there is a tendency to extend the electronic nose term to gas chromatographs and mass spectrometers [11,12], we will use it in the Gardner and Bartlett framework. The array of chemical sensors is the most economical alternative to such instruments, and although it cannot identify the sensed compounds or a human sense of odor [12], it allows the classification of samples based on the signals obtained from volatiles. Besides detecting the volatiles that interact with their chemical coatings, sensor responses depend on their intensity, which is very difficult to evaluate with a human panel, as sensitivities to different odorants vary, with some individuals exhibiting particularly severe anosmia to specific molecules.

Sensing systems, comprising not only the sensors but the sample introduction system, in this case a septum for SPME fiber insertion, a small oven for sample desorption, and the data acquisition and recording system, are designed. Major sensor drawbacks are baseline drift and sensor aging. Baseline drift may be minimized by precisely controlling the temperature and flow rate. Flow injection, which consists of injecting the sample into a carrier flow, allows signal acquisition to take place typically more or less 1 s after the baseline reading. A sensor’s lifetime depends largely on the chosen coatings, and on the temperature and humidity, and may severely limit their use and therefore the number of analyses it can perform.

In this work, samples of office papers with 70 to 80 gsm, bought in markets from Germany, Spain, Italy, France, the Netherlands, Russia, Dubai, and Latin America were analyzed. The manufacturer of each office paper sample was known, but the country of production was not always certain, and in some cases the tree species from which the fibers were extracted was also unknown.

An efficient SPME extraction was used for sampling, after optimizing the time of exposure and the number of paper discs, as well as the nitrogen flow for desorption and the sample carrier for the new e-nose analytical system. The same sampling methodology was used for the Gas Chromatography–Mass Spectrometry (SPME-GC-TOF-MS) analysis of volatiles emitted from paper. pH analyses of the samples were performed as a complementary technique.

The number of sensors in the array was kept to the minimum amount necessary for paper discrimination. A reduced number of sensors should be an array of the most stable highly sensitive coated crystals that the authors succeeded in preparing. In addition, each selected sensor should respond with a different sensitivity pattern to the emitted VOCs. Colinear sensors, meaning sensors showing similar selectivity and related responses, should not be added to the array. These redundant sensors would not be helpful for paper discrimination, and would most probably contribute to increase noise, and lead to overfitting. A high number of sensors is also a more expensive system, with more integration issues, an increased risk in terms of sensor malfunctioning, and increased difficulties in simultaneous data acquisition and handling.

Nevertheless, analysts must always respect the lifetime of SPME fibers and sensors. As raw sensor data are used in this system, without any calibration, which is mandatory and frequent in quantitative analysis, a previously analyzed sample was re-analyzed from time to time to ensure that sensor sensitivities remained the same.

The results allowed discrimination between office paper samples. It was possible to differentiate among paper made from birch, acacia, and *Eucalyptus globulus* trees. Besides wood origin, a few compounds have been identified as possible markers of geographical origin.

## 2. Materials and Methods

### 2.1. Reagents

All reagents used were analytical grade. Coating compounds for piezoelectric quartz crystals were Nafion 117 solution (Nafion), polyvinylpyrrolidone (PVP) and Tetrakis-2-hydroxy ethyl ethylenediamine (THEED) from Fluka (Buchs, Switzerland); Manganese (II) phthalocyanine (Mn-Pht) was from Sigma-Aldrich (Madrid, Spain), and two homemade compounds: chloro [5,10,15,20-tetrakis(pentafluorophenyl)porphyrinato] manganese(III) (Mn(TPFPP)Cl) [13] and tetrabutylammonium Mn (III) polyoxotungstante (TBA-PMnW11) [14].

Solvents used to dissolve coating compounds were chloroform and tetrahydrofuran from Sigma-Aldrich (Madrid, Spain), ethanol from Carlo Erba (Val de Reuil, France) and acetone from VWR (Fontenay-sous-Bois, France).

Nitrogen was Alphagaz from ArLíquido (Algés, Portugal).

SPME fibers and holders were obtained from Supelco (Bellefonte, PA, USA).

### 2.2. Samples

The office paper sheets are part of a personal collection. Office papers with 70 to 80 gsm were bought from markets around the globe, including Germany, Spain, Italy, France, the Netherlands, Russia, Dubai, and Latin America. Papers were not obtained directly through the manufacturers to assure that natural variability was present. Otherwise, samples may have undergone special selection and items could artificially be more homogeneous and standardized. However, this way of gathering samples results in incomplete specifications. Although the manufacturer of each paper is known, the country of production is sometimes uncertain, and for some samples there is no precise information regarding the tree species used as the fiber source. Beyond the lack of information, it is a remarkable collection of items, unlikely to be supplanted.

All samples appeared identical to the naked eye. Table 1 summarizes the sample information. The symbol “?” was used whenever the information was not certain. The samples with complete information are shown in grey.

### 2.3. pH of Paper Samples

pH measurements were performed in the laboratory by cutting approximately 1 g of paper into small pieces and immersing them in 70 mL of distilled water for 1 h. Then, the pH value of the cold extract, unfiltered, was measured with a Crison GLP 22 pH meter with a 5014T combined glass electrode (Barcelona, Spain), following the TAPPI standard 509—Hydrogen ion concentration (pH) of paper extracts: cold extraction method [15,16].

### 2.4. Sampling of VOCs Emitted from Papers

Two different SPME fibers (CAR/PDMS (Carboxen/PDMS) and PDMS (polydimethylsiloxane)) were used because no single coating extracts all compounds well. PDMS (polydimethylsiloxane) fiber is a single-phase, non-polar absorptive liquid-like coating, and, according to the selection guide for Supelco SPME fibers [17], its use is best for non-polar to moderately non-polar compounds, especially mid- to higher-MW semi-volatiles, because its selectivity is mostly based on hydrophobicity (partitioning into PDMS). In terms of volatiles, it is often less sensitive to very volatile, small molecules. CAR/PDMS fiber is a biphasic/porous—CAR (microporous carbon adsorbent) dispersed in PDMS—having a largely adsorptive behavior. It is best for small, highly volatile compounds (low-MW gases/VOCs), often at trace levels. In terms of selectivity, it exhibits strong “molecular sieving” when capturing small molecules, with much higher sensitivity than PDMS for very volatile analytes.

Therefore, for simplicity and to reduce the number of analyses and the cost, only a CAR/PDMS fiber was used for the sensor analytical system. It was the most adequate fiber for the extraction of low-molecular-weight and very volatile analytes [8,17]. Both fibers, CAR/PDMS and PDMS were used in GC-TOF-MS analysis.

Both fibers were initially conditioned according to the manufacturer’s instructions.

The SPME fibers were mounted on SPME holders for manual sampling.

Each A4 office paper sheet was cut with a circular cutter (3.5 cm diameter), yielding 24 circles per sheet. Circles were placed in a clean Petri dish measuring 5 cm in diameter and kept with the lid on in a controlled environment. The temperature was set to 23 °C, and the relative humidity (RH) was set to 35% and maintained for at least 24 h before sampling.

**Figure 1:**
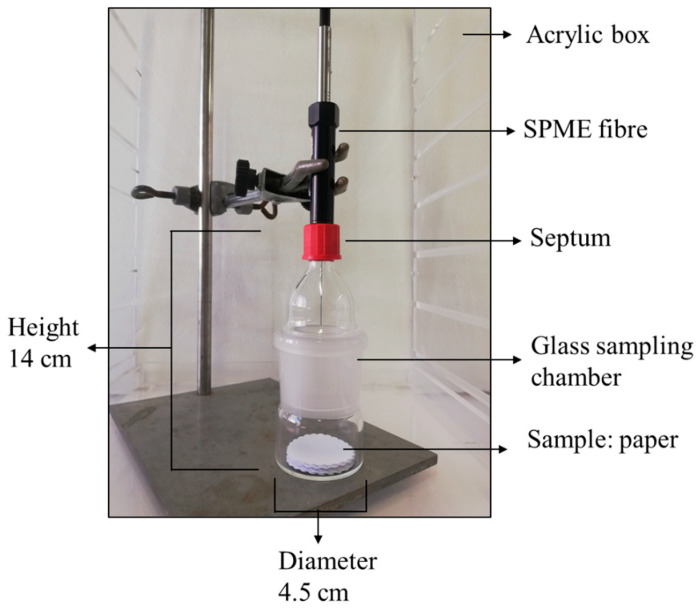
SPME extraction: homemade glass sampling chamber with a paper sample.

Sampling for the sensor analytical system was manual. A chosen number of circles of each sample to be analyzed (22 were used in the analysis after an optimization process) were then placed inside a closed, homemade glass sampling chamber, as shown in Figure 1.

An acrylic desiccator cabinet (Thermo Fisher Nalgene 5371-0180, Waltham, MA USA), equipped with a digital thermometer and humidity reader, was used for housing the glass sampling chamber during SPME sampling to maintain the humidity control after transferring samples from the climatized room to the laboratory sensor room.

The SPME fiber, while retracted into the needle holder, was inserted through the Teflon septum on top of the glass sampling chamber. Afterwards, the fiber was exposed to the sample’s headspace. The exposure time was carefully controlled and, at the end, the SPME fiber was retracted again into the needle holder, removed from the sampling chamber, and inserted into the septum of a clean, closed vial to prevent contamination from the environment during transportation.

For GC-TOF-MS analyses, an autosampler was used. The incubation time was 5 min, the temperature was 40 °C, and the agitation velocity was 250 rpm; the desorption time was 4 min, and the temperature (inlet) was 200 °C.

### 2.5. High-Resolution Gas Chromatography–Mass Spectrometry (HRGCTOF-MS)

The separations were performed using a fused silica capillary column: SLB-5 ms (Supelco, Bellefonte, PA, USA), 60 m × 0.25 mmI.D., film thickness 0.25 µm. The oven temperature was programmed at 35 °C, held for 4 min, increased to 40 °C at 2 °C min^−1^, held for 1 min, increased to 150 °C at 10 °C min^−1^, held for 1 min, and increased to 250 °C at 5 °C min^−1^. Helium was used as carrier gas at a flow rate of 1.0 mL min^−1^. High-resolution gas chromatography–mass spectrometry (HRGC–MS) measurements were performed using a GC-TOF-MS LECO model PEGASUS BT: mode EI+, 70 eV, source temperature 250 °C, interface temperature 250 °C. Full scan mode was used over the range of 30–400 amu (or Da), at a rate of 10 scans s^−1^; the extraction frequency was 30 Hz. As described previously, an autosampler was used. Between analyses, a blank was run to prevent carryover. The analyses were performed in triplicate.

### 2.6. Electronic-Nose

#### 2.6.1. Coating of the Piezoelectric Quartz Crystals

Piezoelectric quartz crystals were 9 MHz AT-cut, HC-45 with gold electrodes (Euroquartz Limited, Crewkerne, UK). The piezoelectric quartz crystals were coated on both sides with solutions of the coating compounds, namely Nafion, TBA-PMnW11, Mn(TPFPP)Cl, THEED, Mn-Pht, and PVP, using drop deposition followed by spreading by a spin-coater Süss Delta 10 BM (SÜSS MicroTec Lithography GmbH, München, Germany) or by spray deposition (BADGER model 200, Badger Air-Brush, Co., Bellwood, IL, USA). Solvents were then allowed to evaporate until the quartz crystal oscillation frequency became stable. The amount of coating was estimated from the difference between the bare crystal’s frequency and the coated crystal’s frequency after solvent evaporation.

#### 2.6.2. Set-Up of the Electronic Nose and Analytical Methodology

**Figure 2:**
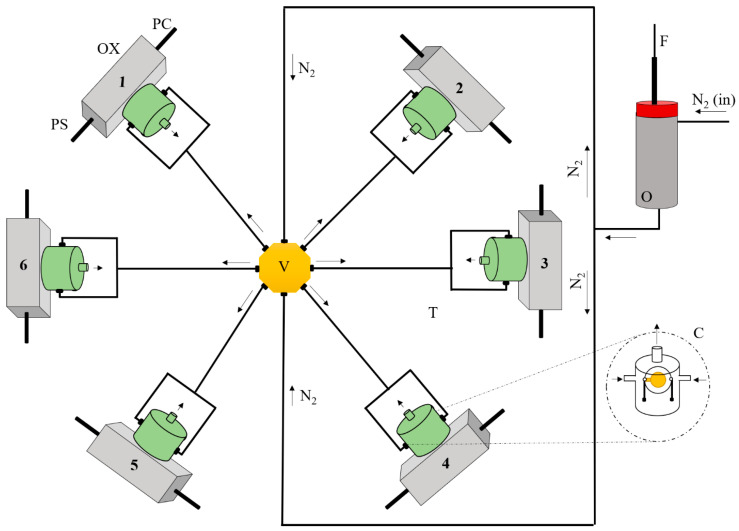
A simplified schematic experimental set-up of the array of six sensors: N_2_—nitrogen flow, F—SPME fiber, O—oven, V—distribution valve, OX—homemade oscillator, PS—power supply, PC—output of frequency of oscillation to a counter and storing data in a personal computer, T—polymeric cell housing the piezoelectric quartz crystal, C—piezoelectric quartz crystal inside the cell.

Figure 2 schematically shows the experimental setup. Compounds from the SPME fiber were thermally desorbed in a homemade oven set to 250 °C. A nitrogen flow carried the desorbed VOCs to the 6 sensors, operating simultaneously. The experimental layout for the e-nose, as well and the PVC piezoelectric quartz crystals cell design, were elsewhere [9].

The first step, the extraction step, consisted of sampling the volatile compounds from papers for 30 min, as described in detail in Section 2.5. After this period, the SPME fiber was retracted and introduced into the pre-heated oven (O). Next, the volatile compounds were thermally desorbed from the SPME fiber and a nitrogen carrier gas (N2) transported them to the distribution valve (V). This valve splits the sample stream among the six (6) piezoelectric crystals, each one with a different chemical coating. Each piezoelectric quartz crystal (C) was housed in a cell (T) and connected to electronic homemade oscillators (OX).

The oscillation frequencies of the sensors were simultaneously monitored and stored on a PC at intervals of 1 s, using a Counter/Timer device PXI 1033 from National Instruments, Austin, TX, USA, and software written in LabView 7.0. Figure S1 in the Supplementary Materials shows the simultaneous frequency recordings from the six sensors. A long baseline shows the frequency stability of all sensors. Their different oscillation frequencies are dictated by the amount and acoustic properties of the coatings. When compounds are released from the SPME fiber, carried by the nitrogen flow, and reach each coated crystal, the frequencies of the six sensors start decreasing simultaneously, as the main flow is equally divided into six streams by the distribution valve (V) and the sensors are equidistant from it.

Due to the continuous nitrogen flow and the reversibility of interactions between coatings and volatile compounds, frequency increases were soon observed, as compounds were swept away and initial frequencies were promptly restored. The difference between the baseline frequency, obtained under pure nitrogen, and the minimum frequency recorded was computed for each sensor.

The SPME fiber (F) was removed from the oven (O) only when the oscillation frequency of all the crystals had returned to baseline values, ensuring the complete recovery of all sensors and complete fiber desorption.

#### 2.6.3. Sampling/Desorption Optimization

Sorption of paper volatiles by SPME and desorption/carrier gas flow for the analysis were optimized using the simplex method. The objective was to maximize the sensors’ responses. Three variables were considered: the number of paper circles used in sampling, the exposure time of the SMPE fiber to the paper sample, and the nitrogen flow rate.

## 3. Results and Discussion

### 3.1. Paper pH

Paper typically contains mainly cellulose, a natural polymer forming long chains (fibers), and can be classified into three main types—rag, acidic, and contemporary (neutral/alkaline)—as a function of their different acidity (pH) and degree of polymerization (DP) [18]. Paper pH depends on pulping and bleaching processes, compound degradation, sizing reagents, and deliberate uses of alkaline stock. Contemporary paper is made from highly processed wood pulp with alkaline reserves. The pH of the analyzed paper samples is presented in Table 2.

As shown in Table 2, the samples had pH values between 9 and 10. The papers were produced between 2018 and 2019 (Table 1). Since the 1980s, to prevent paper degradation, free-acid paper has been produced using alkaline stock [19].

### 3.2. Headspace Solid-Phase Micro-Extraction/Gas Chromatography–Mass Spectrometry (HS-SPME/GC–MS) Analysis

Two types of SPME fibers were used in volatile analyses by HS-SPME/GC-MS. Figures S2 and S3 in the Supplementary Materials show the chromatograms obtained for sample P13 using a CAR/PDMS fiber and a PDMS fiber, respectively. Table 3 describes the main volatile compounds identified in samples using CAR/PDMS-SPME fiber. The VOCs were identified by comparing spectra with (NIST) library spectra and by matching the retention times to those of standards (pure compounds). Tables S1 and S2 (Supplementary Materials) describe the compounds identified in each paper sample using CAR/PDMS and PDMS SPME fibers, respectively.

**Figure 3:**
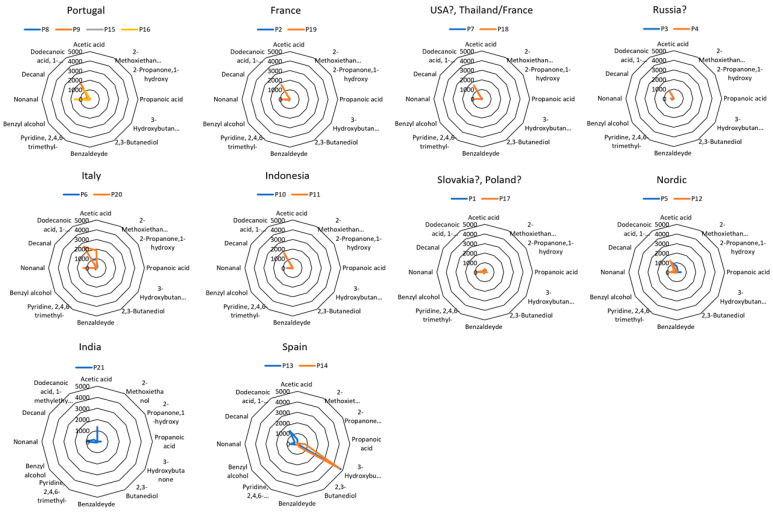
Radial plots of CAR/PDMS-GC-TOF-MS analyses of volatiles identified in paper samples, grouped by country.

Figure 3 shows the volatile profiles of papers analyzed using the CAR/PDMS fiber (samples P1–P21, Table 1) and grouped by country of origin. The scale was the same across all the graphics to facilitate the visualization and comparison of results. Individual sample radial plots, as well as the summary table of all results, are provided in the (Supplementary Materials Table S1 and Figure S4, respectively).

The volatile compounds emitted by office papers are related to wood and to the industrial paper-making process. This means that they should reflect the presence (or not) and relative content of rosin, lignin, cellulose degree of polymerization, carbonyl group content, and paper acidity [19]. The hemicellulose content is higher in hardwood than in softwood. Acetic acid is derived from the degradation of acetylated hemicelluloses from wood through the hydrolysis of acetyl group esters during paper-making technology. If hemicellulose is not acetylated, acetic acid is not a significant volatile product [19,20,21]. During the (paper-making) process, cellulose, the main chemical constituent of wood, degrades into low-molecular-weight organic acids through hydrolytic and oxidative reactions. Acid hydrolysis of cellulose and hemicellulose leads to the formation of reducing alcohol groups, while oxidation leads to the formation of carboxylic acids [19,22,23]. Formic acid, on the other hand, is formed during the microbial fermentation of the sugars present in the wood. Furfural and vanillin are degradation products of lignin and can be considered as its main markers. The presence of alkanes and aldehydes indicates lipid oxidation. The various chemicals involved in paper production may also produce volatile compounds, including alcohols, aldehydes, ketones, carboxylic acids, and hydrocarbons.

As shown in Figure 3, Portuguese paper samples from E. globulus (P8, P9, P15, and P16), produced by the same manufacturer (The Navigator Company, Lisbon, Portugal) but from two different plants (ATF and FIG), exhibited similar volatile composition with predominance of an ester and an aldehyde: dodecanoic acid 1-methylethyl ester (isopropyl dodecanoate) and nonanal. When comparing Portuguese paper profiles with those from E. globulus papers from Spain (samples P13 and P14), differences were observed: a strong signal for 3-hydroxybutanone, a ketone, occurred in the Spanish samples. Spanish paper sample P13 showed very low levels of isopropyl dodecanoate and nonanal compared to the Portuguese papers, compounds that were not detected in the second Spanish paper sample, P14, which instead showed the presence of propanoic acid. Because pulps were also obtained from E. globulus trees, the differences in volatiles may be attributed to differences in the industrial manufacturing processes in Portugal and Spain.

Samples from Italy (P6 and P20) exhibited a volatile profile similar to that of Portuguese papers, but acetic acid was more abundant.

Comparing volatile profiles of papers from France (samples P2 and P19), the USA (sample P7, origin not fully confirmed), Thailand/France (sample P18), Russia (samples P3 and P4, origin not fully confirmed), and Indonesia (samples P10 and P11), all of them have close VOC profiles. As shown in Table 1, the Indonesian papers (samples P10 and P11) are from acacia trees and also show the predominance of isopropyl dodecanoate and nonanal. Both eucalyptus and acacia are sources of hardwood fibers. It is well known that acacia is a strong competitor of eucalyptus in Asia, due to propitious ecological conditions. So, it seems that the wood composition of acacia is similar to that of eucalyptus, and the paper-making technology used in Indonesia is the same as that in Portugal.

Samples P5 and P12, both from Nordic birch pulp, show differences that could arise from differences in paper production by different manufacturers (UPM for P5 and STORAENSO for P12).

The paper sample from India (sample P21) was confirmed to be manufactured from recycled paper. It showed a very different profile, with a prominent amount of acetic acid. Recycled paper is an eco-friendly option for manufacturing paper products, whose production process involves different types of raw materials: pre-consumer waste, post-consumer waste, and sawdust. The increased presence of acetic acid is likely due to both the treatment of the raw material and the production process for recycled paper [23].

Not surprisingly, furfural was not detected in the paper compositions studied, all of which had an alkaline pH.

Concerning mixed papers from Slovakia and Poland, both contained acetic acid and nonanal, although the values obtained were very low compared with those of other samples. From these profiles, no conclusions could be drawn because the wood type is unknown and the country of origin has not been confirmed.

**Figure 4:**
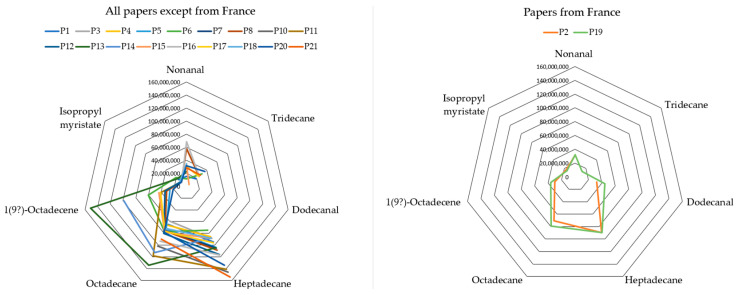
Radial plots of PDMS-GC-TOF-MS main compounds (excluding dodecanoic acid 1-methylethyl ester as it would overwhelm the graphs due to its much higher peak area) for comparison between French papers and papers from other origins.

Papers from France (samples P2 and P19) exhibited a similar volatile profile to that of Portuguese papers also produced from *E. globulus* trees. However, looking at the profiles displayed in Figure 4, obtained with the PDMS fiber, papers from France (P2 and P19) could be distinguished as dodecanal was detected on no other samples.

In summary, SPME-GC-TOF-MS results allowed the identification of the country of origin of paper samples. 3-Hydroxybutanone (acetoin) was identified as a marker for papers produced only in Spain, and dodecanal for papers produced in France. In addition, “Spain and France profiles”, an “India profile” and an “Italy profile” were also identified.

### 3.3. SPME/E-Nose Analysis

#### 3.3.1. Sensors Coatings

Table 4 shows the frequency decrease in the piezoelectric quartz crystals due to coating. The coatings were selected with the understanding that they must physically adsorb the volatile compounds reported to be responsible for the paper aroma. The interaction between the sensor coating and the volatile compounds must not involve covalent bonds, as such interactions would preclude sensor reversibility. Selectivity is not an issue in electronic noses, but different sensitivities to the target compounds among the sensors are required.

#### 3.3.2. Optimization of the E-Nose Methodology

The simplex design methodology was used to optimize the analytical methodology. The objective was to maximize the analytical signal (sensor response). Three variables were expected to have a major effect on the e-nose response, affecting sampling and analysis: (i) the nitrogen flow rate, (ii) the number of paper circles (quantity of paper used for sampling) and (iii) the exposition time of the SPME fiber to volatiles emitted from the paper. The experiments proposed by the Simplex method stopped after the 46th run, upon convergence, indicating that the optimal solution has been found. The optimum objective function was reached with 22 paper circles, 30 min of exposure of the fiber, and a desorption/transporting nitrogen flow rate of 105 mL min^−1^.

#### 3.3.3. Results with the SPME/E-Nose

The headspace extraction of volatiles by SPME from books has been used previously with success, followed by the analysis with a homemade e-nose, which led to book discrimination by paper degradation state [9]. In addition, it helped identify acidic papers made from wood, which were distinguished from those made from cotton or neutral/alkaline wood papers. The e-nose system was changed for the present work, as new sensors needed to be incorporated. Sampling needed to be adapted and experimental conditions optimized. All paper samples were analyzed according to the procedure described in Section 2.6.2.

##### Hierarchical Clustering Analysis (HCA)

HCA is an unsupervised chemometric technique that reveals natural groupings among samples characterized by the values of a set of measured variables. The results were described using a dendrogram, which has a tree-like structure. HCA was performed on 21 samples. For testing purposes, the results from three replicates of acacia P10 (paper from three different sheets from the same ream of paper) and two replicates of recycled P21 were included in the analysis. For all the other samples, only the result of the analysis of 22 circles of the same sheet was included to keep the dendrogram as simple as possible.

**Figure 5:**
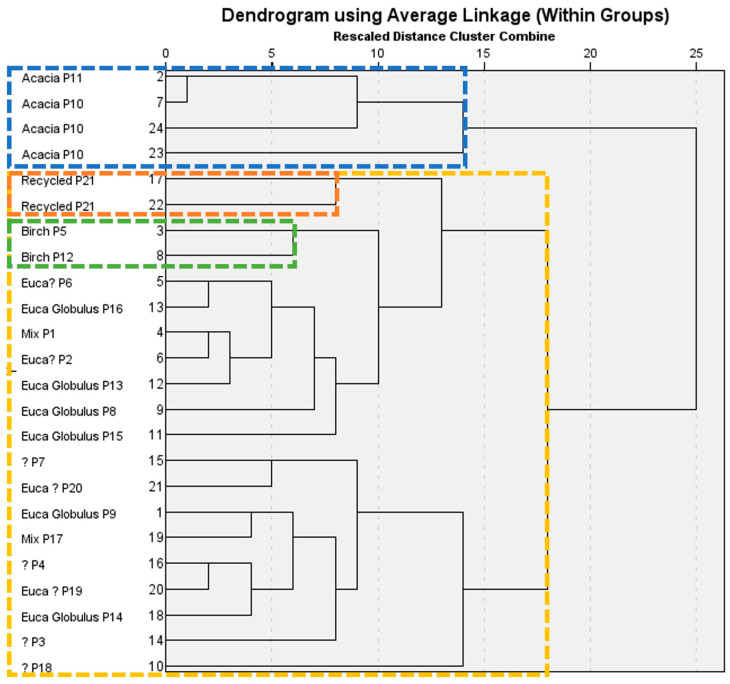
Cluster dendrogram of paper samples manufactured with different types of wood.

Cluster analysis was conducted using hierarchical agglomerative clustering with average linkage (within groups). Euclidean distance was used as the dissimilarity metric to quantify similarities/differences among samples, and the resulting clustering can be visualized in Figure 5.

At a distance level of 18, all the samples can be clustered into two groups (see Figure 5). The first cluster, I (blue line), consists of acacia samples (P10 and P11), and the second cluster, II (yellow line), consists of all the remaining samples. This second cluster aggregates all E. Globulus, birch, mix, and recycled papers, and includes all the remaining samples without information about the tree of origin. Replicates are together, as expected.

The findings are quite promising and suggest a significant potential for future research on identifying the origin of office paper sheets.

**Figure 6:**
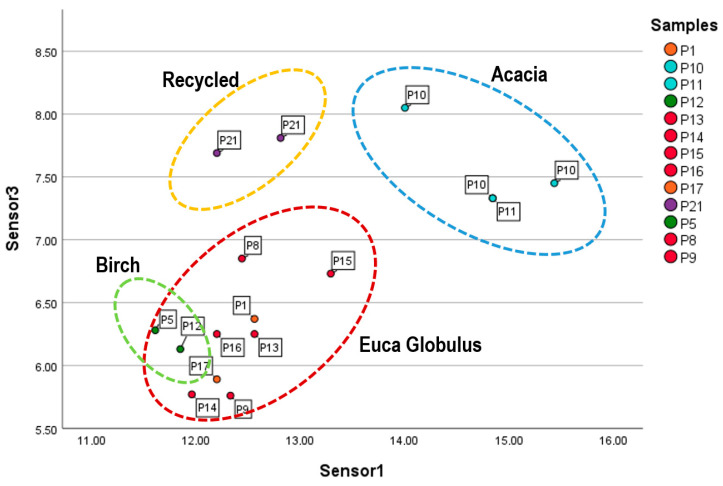
Plot of the response of sensor 3 vs. sensor 1, excluding all the dubious samples.

Figure 6 shows a plot of the frequency decreases observed for sensor 1 vs. sensor 3 for samples with known wood type. It can be observed that acacia paper elicits the strongest responses from both sensors 1 and 3, whereas recycled paper elicits the highest responses from sensor 3 but lower responses from sensor 1. Birch paper samples exhibit low responses from both sensors 1 and 3, while E. Globulus and mix papers are also characterized by low responses from sensor 3 but higher responses from sensor 1. These findings suggest that the two sensors can differentiate among various wood types used in paper production.

## 4. Conclusions

The research presents the results of two methods of analysis of volatiles emitted by office papers. Both begin with the extraction and preconcentration of the volatiles at room temperature, which were then desorbed from the SPME fiber and analyzed by a GC-TOF-MS instrument and a homemade electronic nose based on coated piezoelectric quartz crystals. Two sensors of the array, coated with PVP and THEED, respectively, enabled the discrimination of papers based on the tree used to obtain the wood pulp, as well as the identification of recycled paper. GC-TOF-MS analysis enabled the identification of the VOCs, and to find a few possible markers of the paper’s geographic origin. More samples should be included in a future study to validate these findings and build a robust model for paper identification, which could be useful for paper traceability for various purposes, including conservation, cultural heritage, forgery detection and other forensic applications, audits, the printing of official documents and new book editions.

## Figures and Tables

**Table 1:** Characterization of office paper samples.

ID	Type of Wood *	Paper Grammage (g)	Market	Manufacturer	Country of Origin *	Year of Production
P1	Mix	80	France	MONDI	Slovakia?	2019
P2	*E. globulus*?	80	Germany	CLAIRE FONTAINE	France	2019
P3	-	80	Russia	INTERNATIONAL PAPER	Russia?	2018
P4	-	80	Russia	MONDI	Russia?	2018
P5	Birch	70	Germany	UPM	Nordic	2019
P6	*E. globulus*?	75	Italy	BURGO	Italy	2019
P7	-	80	Germany	INTERNATIONAL PAPER	USA?	2018
P8	*E. globulus*	75	Portugal	THE NAVIGATOR COMPANY (ATF)	Portugal	2019
P9	*E. globulus*	70	Portugal	THE NAVIGATOR COMPANY (FIG)	Portugal	2019
P10	Acacia	70	Germany	APRIL	Indonesia	2019
P11	Acacia	80	Germany	APRIL	Indonesia	2019
P12	Birch	80	Germany	STORAENSO	Nordic	2018
P13	*E. globulus*	80	Spain	TORRAS PAPEL	Spain	2019
P14	*E. globulus*	80	Spain	ZINCUÑAGA	Spain	2019
P15	*E. globulus*	80	Portugal	THE NAVIGATOR COMPANY	Portugal	2019
P16	*E. globulus*	80	Portugal	THE NAVIGATOR COMPANY (FIG)	Portugal	2019
P17	Mix	80	-	ARTIC PAPER	Poland?	2018
P18	-	75	-	AA	Thailand/France	2018
P19	*E. globulus*?	80	France	CLAIRE FONTAINE	France	2019
P20	*E. globulus*?	80	Italy	FEDRIGONI	Italy	2019
P21	Recycled?	80	Holland	TNPL	India	2019

* Whenever information is not confirmed, an interrogation mark “?” is placed next to data. Information not known is marked with “-”.

**Table 2:** pH values measured for each paper according to TAPPI standard 509 [hydrogen ion concentration (pH) of paper extracts—cold extraction method]. pH values are an average of 3 replicates.

Sample	pH *	Sample	pH *	Sample	pH *
P1	9.04 ± 0.02	P8	9.92 ± 0.01	P15	9.54 ± 0.01
P2	9.05 ± 0.01	P9	9.92 ± 0.01	P16	10.00 ± 0.02
P3	9.42 ± 0.01	P10	9.44 ± 0.00	P17	9.39 ± 0.00
P4	9.62 ± 0.01	P11	9.45 ± 0.00	P18	10.15 ± 0.01
P5	10.08 ± 0.00	P12	9.18 ± 0.01	P19	9.60 ± 0.02
P6	9.42 ± 0.02	P13	9.92 ± 0.01	P20	9.67 ± 0.00
P7	9.01 ± 0.01	P14	9.82 ± 0.00	P21	9.71 ± 0.02

* Values expressed as mean ± standard deviation obtained from three measurements per replicate.

**Table 3:** Main VOCs identified in paper samples by HS-SPME-GC-MS using CAR/PDMS fiber.

Peak Number	Compound Identification
**1**	acetic acid
**2**	2-methoxyethanol
**3**	1-hydroxy-2-propanone
**4**	propanoic acid
**5**	3-hydroxybutanone
**6**	2,3-butanediol
**7**	benzaldehyde
**8**	2,4,6-trimethylpyridine
**9**	benzyl alcohol
**10**	nonanal
**11**	decanal
**12**	dodecanoic acid 1-methylethyl ester (isopropyl dodecanoate)

**Table 4:** Frequency decreases due to coating (kHz) for the six piezoelectric quartz crystals used on the sensor array.

Coatings	Frequency Decreases Due to Coating (kHz)
PVP	63
Mn-Pht	69
THEED	25
POM	35
Mn(TPFPP)Cl	68
Nafion	58

## Data Availability

The original contributions presented in this study are included in the article/Supplementary Material. Further inquiries can be directed to the corresponding author.

## Supplementary Materials

The following supporting information can be downloaded at https://www.mdpi.com/article/10.3390/s26072049/s1: Figure S1. Typical electronic nose response to desorbed volatile compounds from paper; Figure S2. Chromatogram of sample P13 obtained with a CAR/PDMS fiber; Figure S3. Chromatogram of sample P13 obtained with a PDMS fiber; Table S1. GC-TOF-MS compounds identification in papers from P1 to P21, using a CAR/PDMS fiber; Table S2. GC-TOF-MS compounds identification in papers from P1 to P21, using a PDMS fiber; Figure S4. Profile of volatile compounds obtained by SPME CAR-PDMS/GC-MS for each tested paper sample.
