# Supplementary material for: GC-MS and E-Nose Analysis of Office Paper: Discriminating Paper Origin Using Multivariate Analysis
Source: Sensors (Basel). 2026 Mar 25;26(7):2049. doi: 10.3390/s26072049 (PMC13074344; doi:10.3390/s26072049)
Supplement: Supplementary file 1 [file sensors-26-02049-s001.zip › sensors-4176972-supplementary.pdf]

SUPPLEMENTARY MATERIAL

GC-MS and e-nose analysis of office paper: discriminating paper origin using multivariate analysis

Marta I.S. Veríssimo[a], Elvira Gaspar[b], M. Teresa S. R. Gomes[c*]

[a] Department of Chemistry, University of Aveiro, 3810-193 Aveiro, Portugal

[b] LAQV-REQUIMTE, Department of Chemistry, Faculty of Science and Technology, New University of Lisbon, Quinta da Torre, 2825-114 Caparica, Portugal

[*c]Centre of Environmental and Marine Studies (CESAM), Department of Chemistry, University of Aveiro, 3810-193 Aveiro, Portugal

* mtgomes@ua.pt

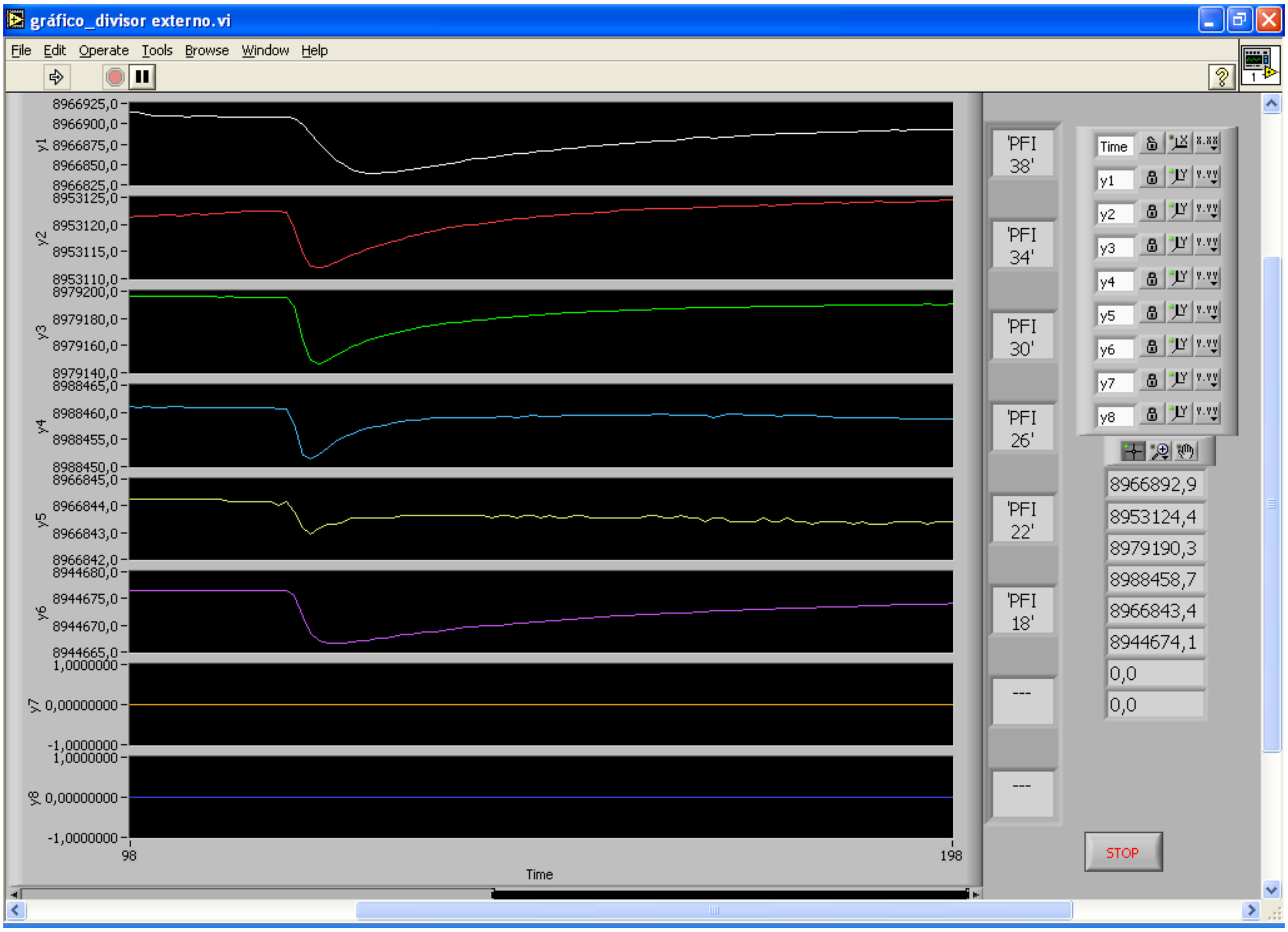

Figure S1. Typical electronic nose response to desorbed volatile compounds from paper. The first six windows show the frequency plots (one for each sensor) over time. Frequency decreases (negative peaks) were produced following the thermal desorption of compounds from the SPME fibre.

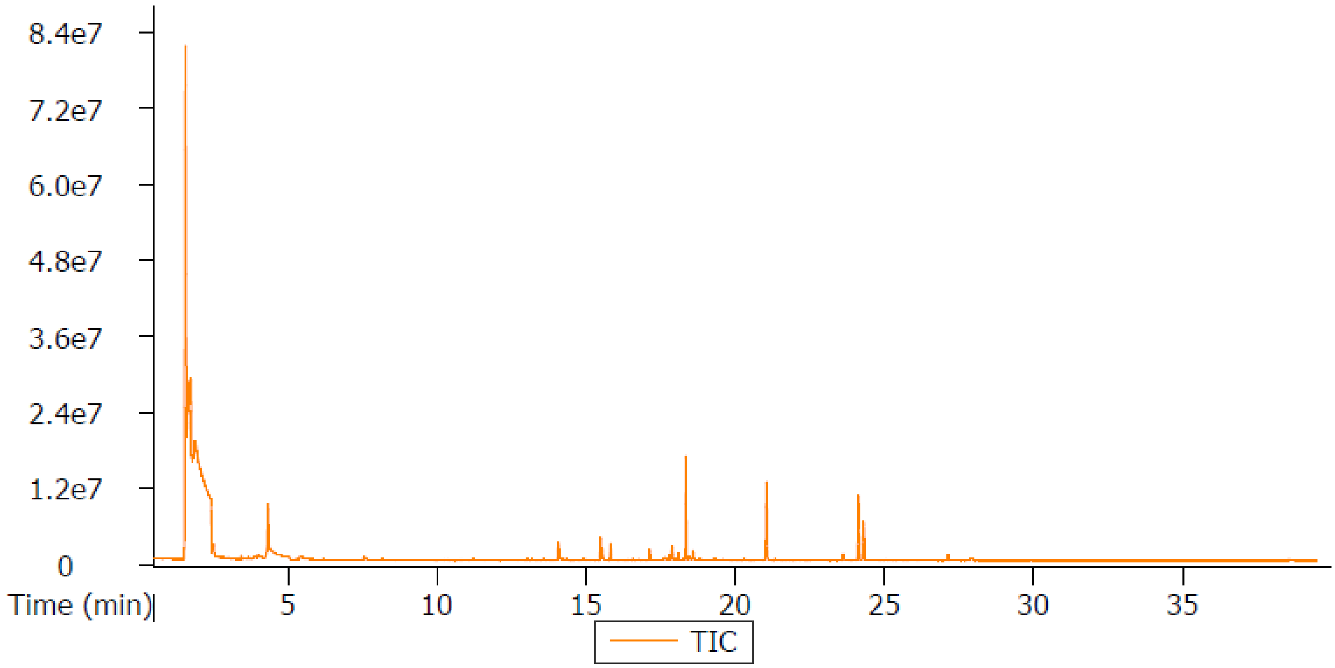

Figure S2. Chromatogram of sample P13 obtained with a CAR/PDMS fibre

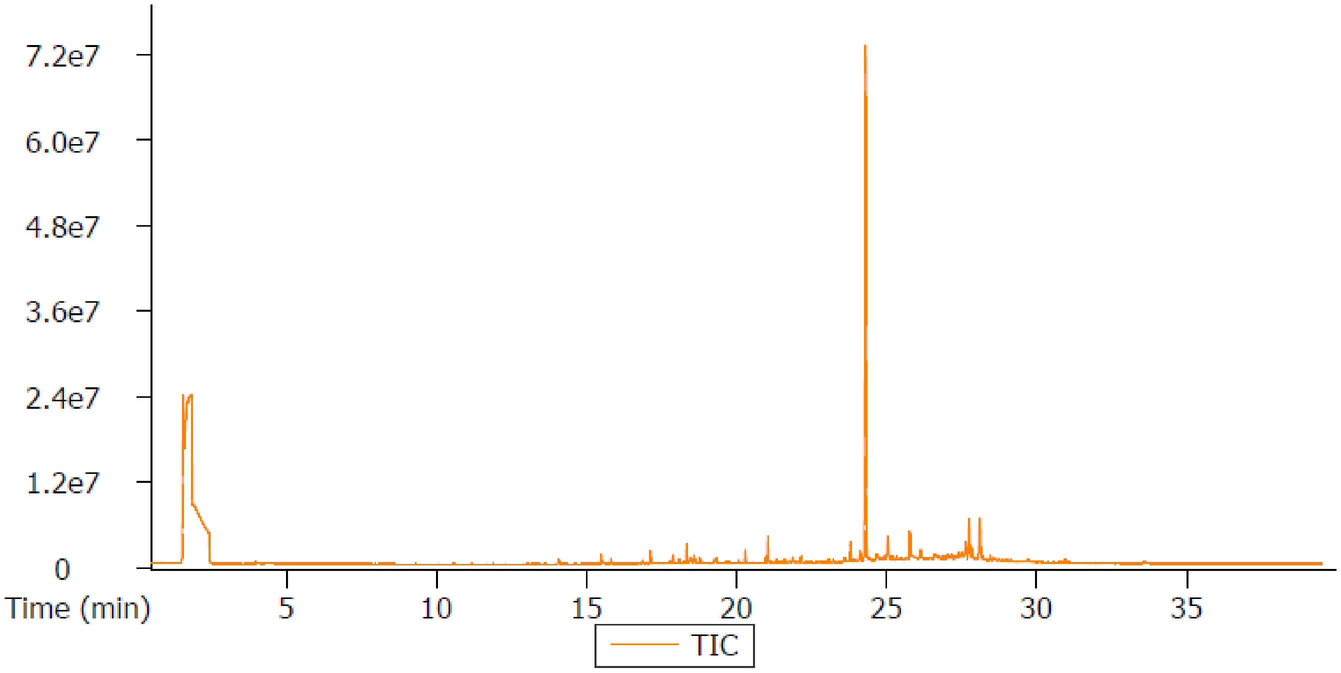

Figure S3. Chromatogram of sample P13 obtained with a PDMS fibre

Table S1 – GC-TOF-MS compounds identification in papers from P1 to P21, using a CAR/PDMS fibre.

CAR/PDMS												
**Sample**	**Acetic acid (2.534 min)**	**2-Methoxy ethanol (3.02 min)**	**1-Hydroxy-2-propanone (3.470 min)**	**Propanoic acid (3.842 min)**	**3-Hydroxy butanone (4.336 min)**	**2,3-Butanediol (6.907 min)**	**Benzaldehyde (12.721 min)**	**2,4,6-trimethyl-pyridine (13.329 min)**	**Benzyl alcohol (14.247 min)**	**Nonanal (15.492 min)**	**Decanal (17.140 min)**	**1-Methylethyl ester dodecanoic acid (24.295 min)**
P1	20.926.243	28.041.655							6.627.922	12.141.774		21.894.673
P2	4.320.139	4.809.410						14.136.233	6.503.871			43.692.395
P3							3.610.770		19388490			57.863.638
P4							3.928.117		6.721.730	30.232.907		88.129.097
P5	60.981.675	21.355.778		44.537.794			3.065.774		3.432.469	31.650.350		126.422.223
P6	86.403.365	19.340.873	11.864.416				5.374.187	16.712.052	5.880.346	29.137.044		106.495.496
P7			8.350.741				3.964.481		7.672.560			124.574.152
P8	3.655.474	6.810.800					4.860.341			100.677.040		88.069.178
P9	4.532.604			2.174.066			5.235.309		9.234.946	86.219.550	27.838.990	188.855.719
P10							3.194.426		6.781.734	58.675.825		152.462.136
P11				3.869.542			5.405.281		9.650.473	52.293.924		182.224.490
P12	8.612.706	3.170.422					4.894.627		12.796.413	72.435.738	30.641.069	138.060.071
P13	41.447.770		12.297.195		480.027.066				10.959.794	65.933.464	27.688.377	141.844.303
P14			11.329.714	73.878.096	462.601.454	30.168.411			8.912.077			
P15							5.273.552		17.751.537	106.135.813	25.309.103	121.900.877
P16							4.799.888		12.754.909	159.123.639		122.960.001
P17	34.564.614			24.721.814			6.236.286	18.833.762		89.371.718	23.590.175	
P18										80.660.588		163.105.361
P19							3.943.476		11.593.348	83.069.871		172.756.478
P20	184.266.322	5.293.483					4.830.303			137.441.595		256.553.982
P21	132.965.593	4.958.799		35.183.647			5.774.654		11.693.970	94.187.555	35.889.360	

Table S2 – GC-TOF-MS compounds identification in papers from P1 to P21, using a PDMS fibre.

									PDMS												
Sample	Nonanal (15.489 min)	Dodecane (17.000 min)	Tridecane (18.478 min)	Tetradecane (20.071 min)	Dodecanal (20.292 min)	5,9-Undecadien-2-one,6,10-[illegible]	Pentadecane (21.876 min)	Tridecanal (22.15 min)	1-Methylethyl ester dodeca-noic acid, (24.295 min)	n-Hexyl salicylate (25.506 min)	Heptadecane (25.776 min)	2-Dodecenal (26.144 min)	Heptadecane, 4-methyl (26.898 min)	Heptadecane, 2-methyl (27.022 min)	Heptadecane, 3-methyl (27.183 min)	3(?)-Octadecene * (27.646 min)	Octadecane (27.755 min)	1(9?)-Octadecene * (28.104 min)	1-Methylethyl ester tetradecanoic [illegible]	Nonadecane (29.700 min)	Methyl ester hexadecanoic acid,
P1	15.075.580	trace	18.807.212	13.633.801		41.787.969	9.404.581		2.218.801.505		94.472.684		14.396.438	16.130.601	12.655.281	14.252.610	65.142.772		16.142.772	16.190.284	4.957.807
P2	31.739.023			14.511.918	32.395.413	16.582.928	15.328.925		2.622.489.170		89.393.909			17.527.259		16.343.342	70.238.335	29.902.217	16.079.727		3.447.229
P3			14.066.365	10.144.116		18.974.956	13.333.528		2.778.049.568	13.070.155	88.390.166		14.137.288	16.802.835			59.452.725		17.207.390		9.791.040
P4			22.207.872	14.945.526		17.178.918	18.250.297		2.507.691.455		85.719.745			16.003.863		15.105.199	66.634.569		11.681.359	14.885.883	7.874.313
P5		2.641.110	32.211.043	13.196.203			10.111.707		3.032.754.449		103.553.233			19.115.426	13.248.590		72.315.741	26.270.397		15.403.573	5.039.916
P6	11.382.712	2.239.498	30.765.544	10.360.096		10.163.934	11.816.263		2.108.033.143		74.864.932			15.670.854	11.230.866	37.415.383	77.280.518	60.486.097	21.021.706	13.146.172	8.851.560
P7		3.267.095	33.628.851	11.540.640		30.879.271	17.202.100		2.827.386.537		105.443.253			20.348.857	14.083.034	22.815.966	75.344.456		15.529.459	16.715.351	5.077.177
P8	57.506.847	3.715.894	24.537.794	10.899.501			32.645.537		1.446.219.754		108.731.115			19.040.483		12.851.669	76.325.530	31.361.228	9.943.281		3.356.343
P9	34.712.863	1.470.900	14.518.533	10.522.630		21.437.538	22.588.163		2.793.117.868		105.565.260			21.665.279	16.849.252	20.649.101	82.579.676	35.154.897			4.337.695
P10	27.221.721			11.128.310		20.735.835	22.616.396		2.183.959.567		146.343.452			22.147.076	14.408.260		101.482.267				3.914.340
P11		1.873.597	22.546.186	12.556.711		17.344.084	16.840.160	35.961.732	2.507.642.060		141.087.364			21.598.795	15.031.190	26.482.781	118.153.657	39.918.528	10.857.871	18.912.611	2.846.002
P12		2.493.408	16.966.109	8.122.726		14.569.714	21.729.676		1.792.966.646		116.344.432			21.223.962	15.062.919	18.613.877	79.855.004	33.869.753	11.282.191	16.134.294	
P13	22.607.208	3.332.096		8.577.673		16.890.529	16.393.972		1.557.731.212		104.510.086			17.591.998	16.006.094	76.455.804	134.314.564	151.422.128	18.437.946		2.704.615
P14		2.216.843	24.366.565	9.082.960		17.647.141	15.873.849		278.960.061		87.676.795	104.770.566		27.677.132	14.003.936	54.483.781	112.751.189	98.929.140		14.729.420	
P15	33.388.576	1.990.331	4.745.242	9.338.514			21.127.293		1.080.643.985		88.755.057			16.793.793			78.291.853	43.450.394		13.457.427	
P16	68.010.402	2.059.105	25.081.664	9.896.090			26.751.804		1.562.241.700		116.835.886			21.037.396	15.289.941	26.574.711	93.141.800		9.859.751	16.367.629	3.707.686
P17	30.005.635	2.214.192	24.059.682	14.610.729			20.566.007		2.787.986.982		95.794.913			18.739.368		24.126.798	76.750.618	41.240.884		16.126.770	5.314.581
P18	34.278.205			7.938.911		9.938.373	9.769.876		2.243.692.552		88.406.387			17.207.878	12.197.331	16.310.5666	72.048.092		12.322.223	15.575.719	3.251.452
P19	31.950.106		13.084.908	11.347.038	44.654.332	17.470.076	20.340.393	10.304.508	2.450.823.530		89.788.784			18.866.300	12.411.802		79.171.120	36.191.507	14.914.851	18.568.700	
P20	31.447.337	4.371.016	35.925.289	20.584.453		17.269.029	27.219.265		3.265.128.273		134.413.861		18.119.879	22.883.094	16.047.500		78.399.322	21.315.321	10.615.049	15.181.057	4.903.777
P21	28.571.323	2.209.271	27.458.831	31.067.970		36.823.457	38.322.662		4.340.889.227		154.650.304		0	26.779.577	19.148.683	24.501.544	89.872.857				

*The double bond position was not confirmed.

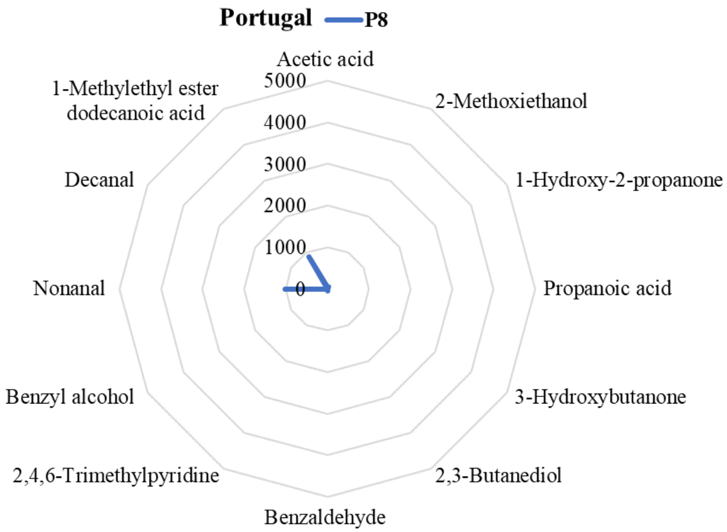
Portugal
P8
Acetic acid
2-Methoxiethanol
1-Hydroxy-2-propanone
Propanoic acid
3-Hydroxybutanone
2,3-Butanediol
Benzaldehyde
2,4,6-Trimethylpyridine
Benzyl alcohol
Nonanal
Decanal
1-Methylethyl ester dodecanoic acid
5000
4000
3000
2000
1000
0

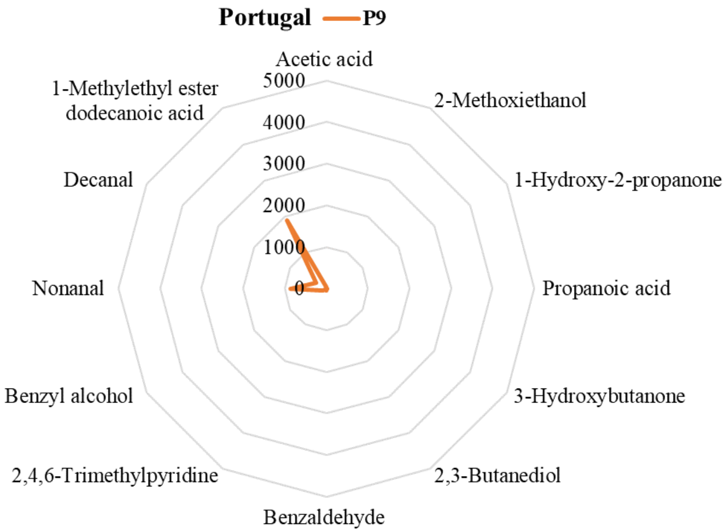
Portugal
P9
Acetic acid
2-Methoxiethanol
1-Hydroxy-2-propanone
Propanoic acid
3-Hydroxybutanone
2,3-Butanediol
Benzaldehyde
2,4,6-Trimethylpyridine
Benzyl alcohol
Nonanal
Decanal
1-Methylethyl ester dodecanoic acid
5000
4000
3000
2000
1000
0

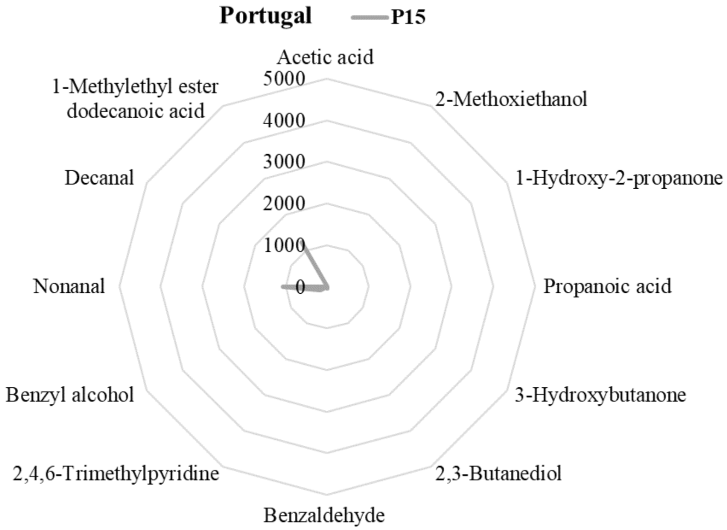
Portugal
P15
Acetic acid
2-Methoxiethanol
1-Hydroxy-2-propanone
Propanoic acid
3-Hydroxybutanone
2,3-Butanediol
Benzaldehyde
2,4,6-Trimethylpyridine
Benzyl alcohol
Nonanal
Decanal
1-Methylethyl ester dodecanoic acid
5000
4000
3000
2000
1000
0

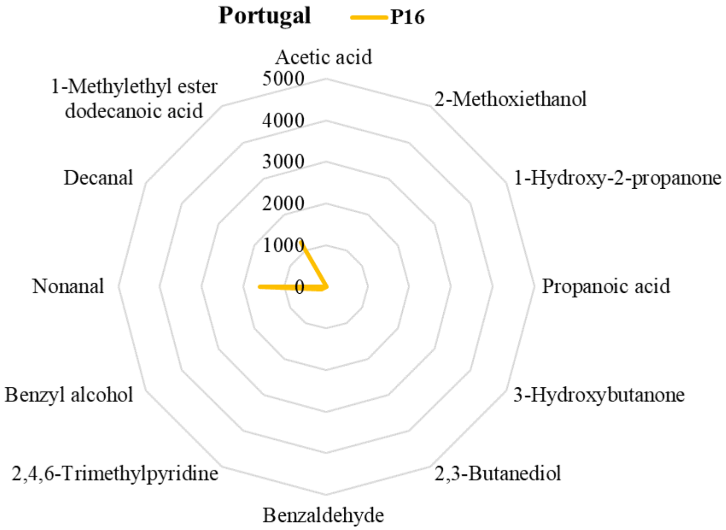
Portugal
P16
Acetic acid
2-Methoxiethanol
1-Hydroxy-2-propanone
Propanoic acid
3-Hydroxybutanone
2,3-Butanediol
Benzaldehyde
2,4,6-Trimethylpyridine
Benzyl alcohol
Nonanal
Decanal
1-Methylethyl ester dodecanoic acid
5000
4000
3000
2000
1000
0

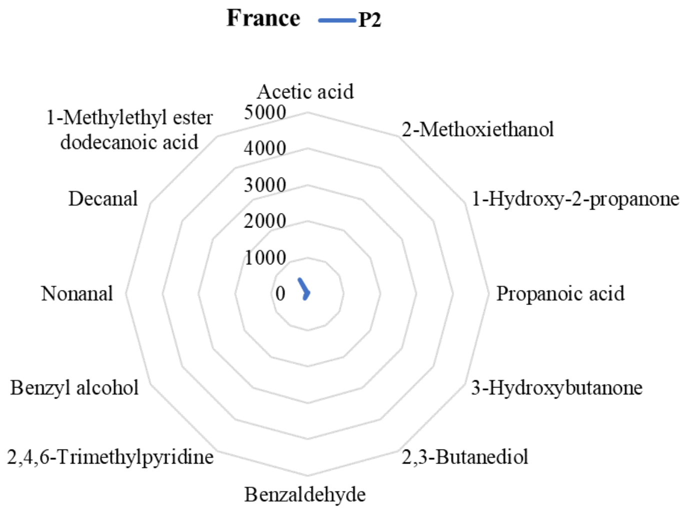
France
P2
Acetic acid
2-Methoxiethanol
1-Hydroxy-2-propanone
Propanoic acid
3-Hydroxybutanone
2,3-Butanediol
Benzaldehyde
2,4,6-Trimethylpyridine
Benzyl alcohol
Nonanal
Decanal
1-Methylethyl ester dodecanoic acid
5000
4000
3000
2000
1000
0

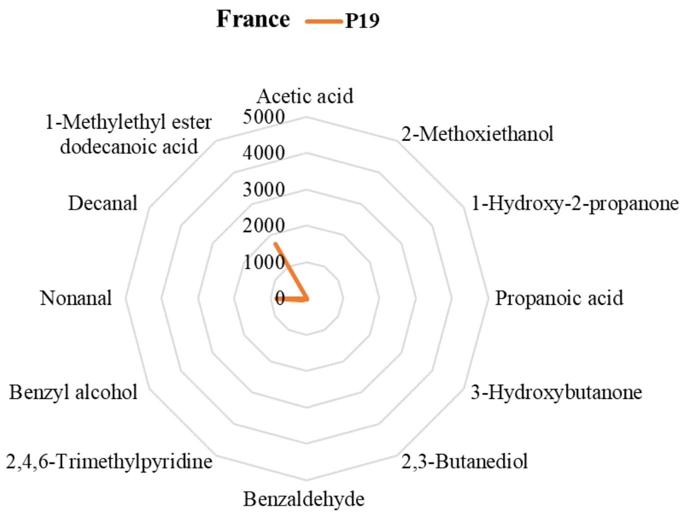
France
P19
Acetic acid
2-Methoxiethanol
1-Hydroxy-2-propanone
Propanoic acid
3-Hydroxybutanone
2,3-Butanediol
Benzaldehyde
2,4,6-Trimethylpyridine
Benzyl alcohol
Nonanal
Decanal
1-Methylethyl ester dodecanoic acid
5000
4000
3000
2000
1000
0

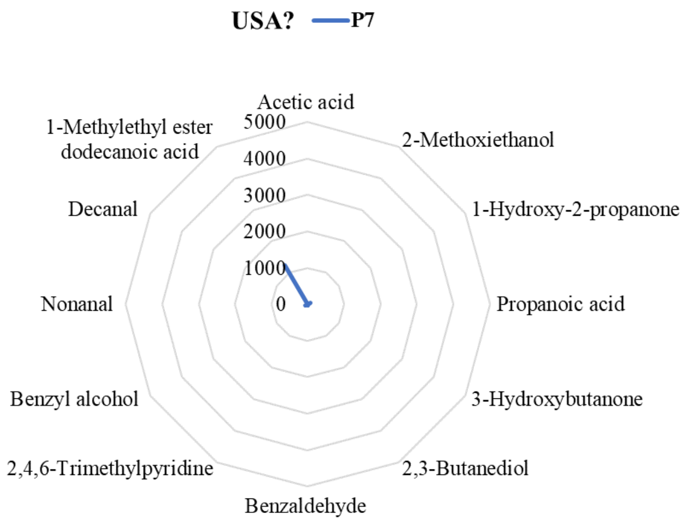
USA?
P7
Acetic acid
2-Methoxiethanol
1-Hydroxy-2-propanone
Propanoic acid
3-Hydroxybutanone
2,3-Butanediol
Benzaldehyde
2,4,6-Trimethylpyridine
Benzyl alcohol
Nonanal
Decanal
1-Methylethyl ester dodecanoic acid
5000
4000
3000
2000
1000
0

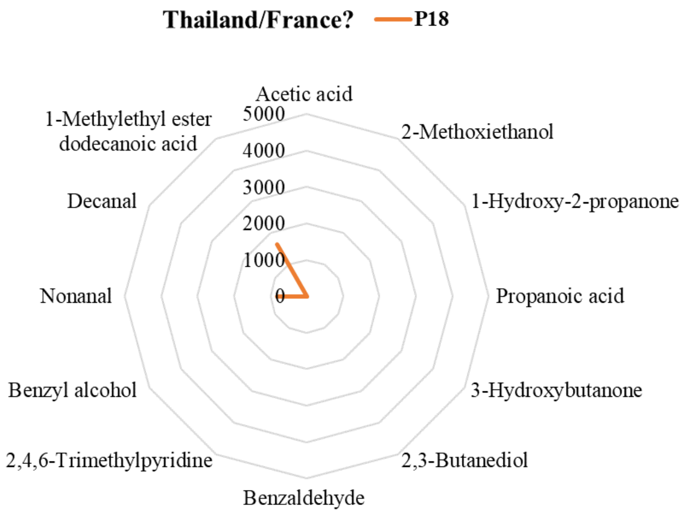
Thailand/France?
P18
Acetic acid
2-Methoxiethanol
1-Hydroxy-2-propanone
Propanoic acid
3-Hydroxybutanone
2,3-Butanediol
Benzaldehyde
2,4,6-Trimethylpyridine
Benzyl alcohol
Nonanal
Decanal
1-Methylethyl ester dodecanoic acid
5000
4000
3000
2000
1000
0

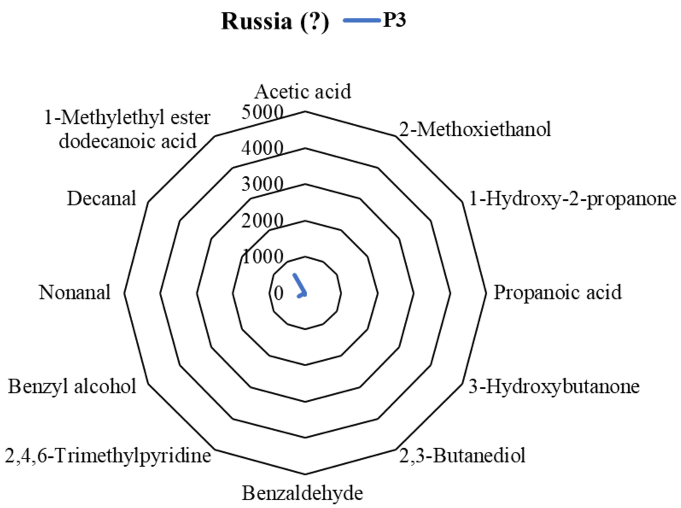
Russia (?)
P3
Acetic acid
2-Methoxiethanol
1-Hydroxy-2-propanone
Propanoic acid
3-Hydroxybutanone
2,3-Butanediol
Benzaldehyde
2,4,6-Trimethylpyridine
Benzyl alcohol
Nonanal
Decanal
1-Methylethyl ester dodecanoic acid
5000
4000
3000
2000
1000
0

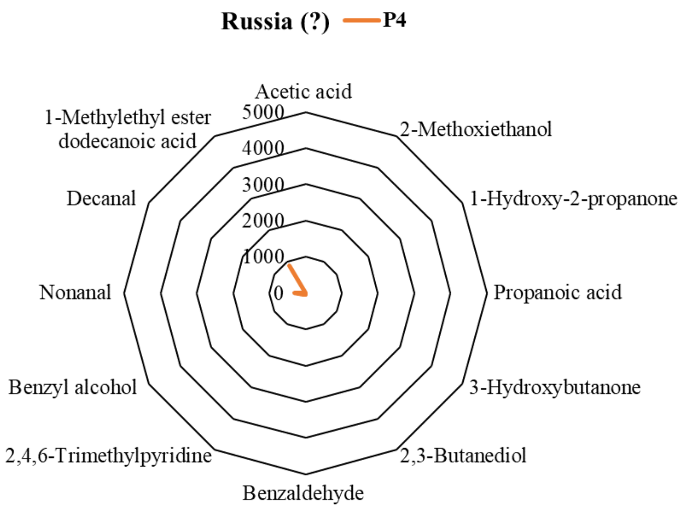
Russia (?)
P4
Acetic acid
2-Methoxiethanol
1-Hydroxy-2-propanone
Propanoic acid
3-Hydroxybutanone
2,3-Butanediol
Benzaldehyde
2,4,6-Trimethylpyridine
Benzyl alcohol
Nonanal
Decanal
1-Methylethyl ester dodecanoic acid
5000
4000
3000
2000
1000
0

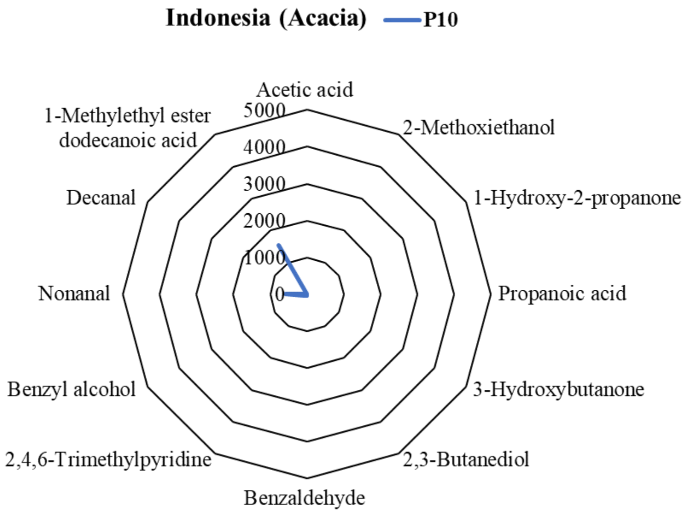
Indonesia (Acacia)
P10
Acetic acid
2-Methoxiethanol
1-Hydroxy-2-propanone
Propanoic acid
3-Hydroxybutanone
2,3-Butanediol
Benzaldehyde
2,4,6-Trimethylpyridine
Benzyl alcohol
Nonanal
Decanal
1-Methylethyl ester dodecanoic acid
5000
4000
3000
2000
1000
0

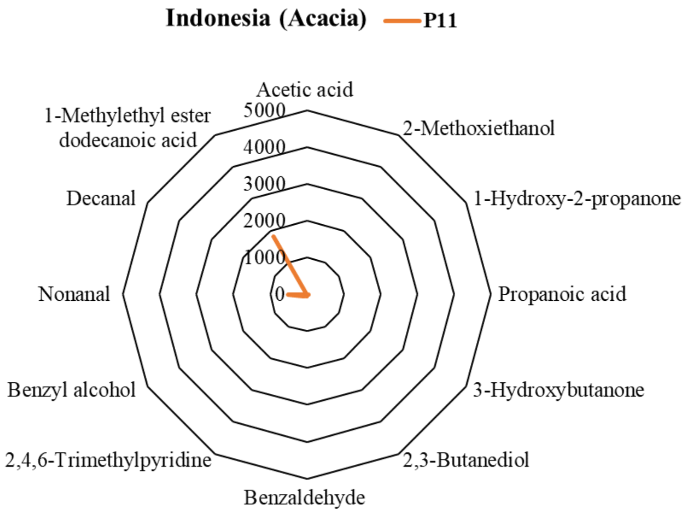
Indonesia (Acacia)
P11
Acetic acid
2-Methoxiethanol
1-Hydroxy-2-propanone
Propanoic acid
3-Hydroxybutanone
2,3-Butanediol
Benzaldehyde
2,4,6-Trimethylpyridine
Benzyl alcohol
Nonanal
Decanal
1-Methylethyl ester dodecanoic acid
5000
4000
3000
2000
1000
0

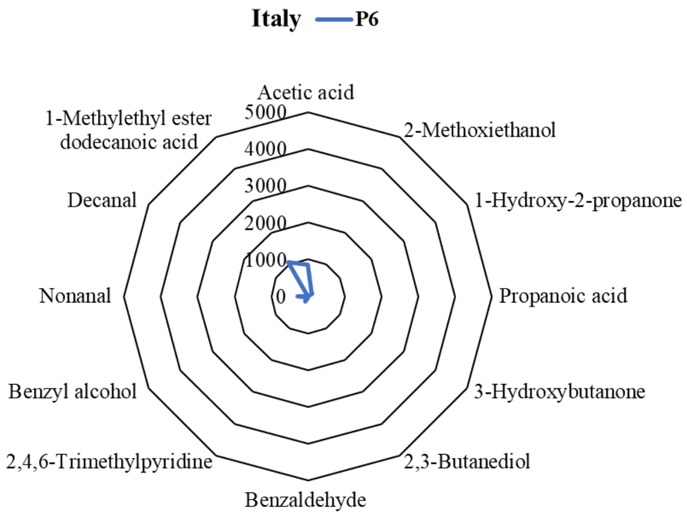
Italy
P6
Acetic acid
2-Methoxiethanol
1-Hydroxy-2-propanone
Propanoic acid
3-Hydroxybutanone
2,3-Butanediol
Benzaldehyde
2,4,6-Trimethylpyridine
Benzyl alcohol
Nonanal
Decanal
1-Methylethyl ester dodecanoic acid
5000
4000
3000
2000
1000
0

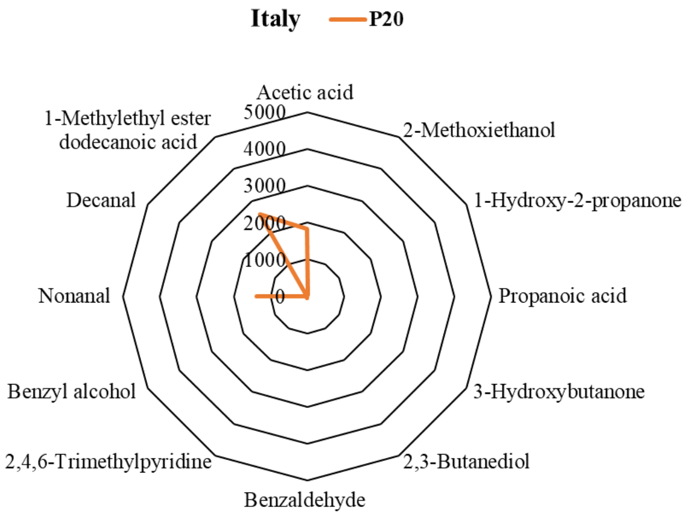
Italy
P20
Acetic acid
2-Methoxiethanol
1-Hydroxy-2-propanone
Propanoic acid
3-Hydroxybutanone
2,3-Butanediol
Benzaldehyde
2,4,6-Trimethylpyridine
Benzyl alcohol
Nonanal
Decanal
1-Methylethyl ester dodecanoic acid
5000
4000
3000
2000
1000
0

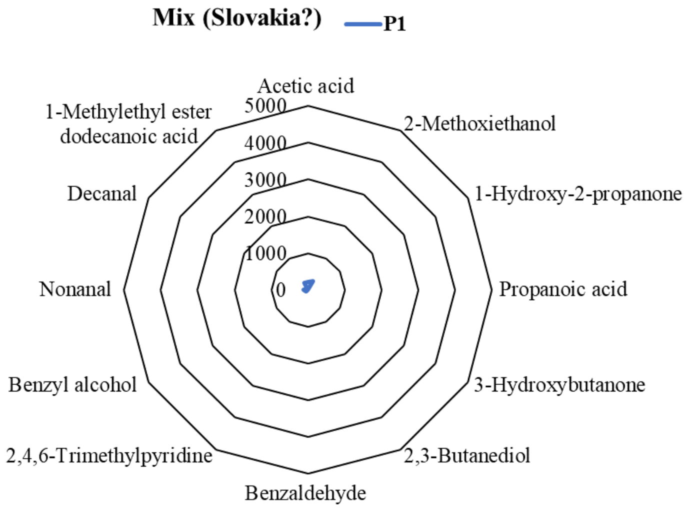
Mix (Slovakia?)
P1
Acetic acid
2-Methoxiethanol
1-Hydroxy-2-propanone
Propanoic acid
3-Hydroxybutanone
2,3-Butanediol
Benzaldehyde
2,4,6-Trimethylpyridine
Benzyl alcohol
Nonanal
Decanal
1-Methylethyl ester dodecanoic acid
5000
4000
3000
2000
1000
0

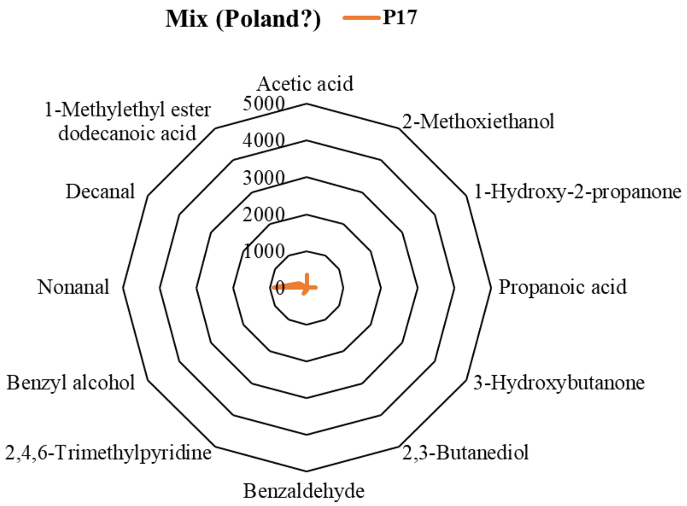
Mix (Poland?)
P17
Acetic acid
2-Methoxiethanol
1-Hydroxy-2-propanone
Propanoic acid
3-Hydroxybutanone
2,3-Butanediol
Benzaldehyde
2,4,6-Trimethylpyridine
Benzyl alcohol
Nonanal
Decanal
1-Methylethyl ester dodecanoic acid
5000
4000
3000
2000
1000
0

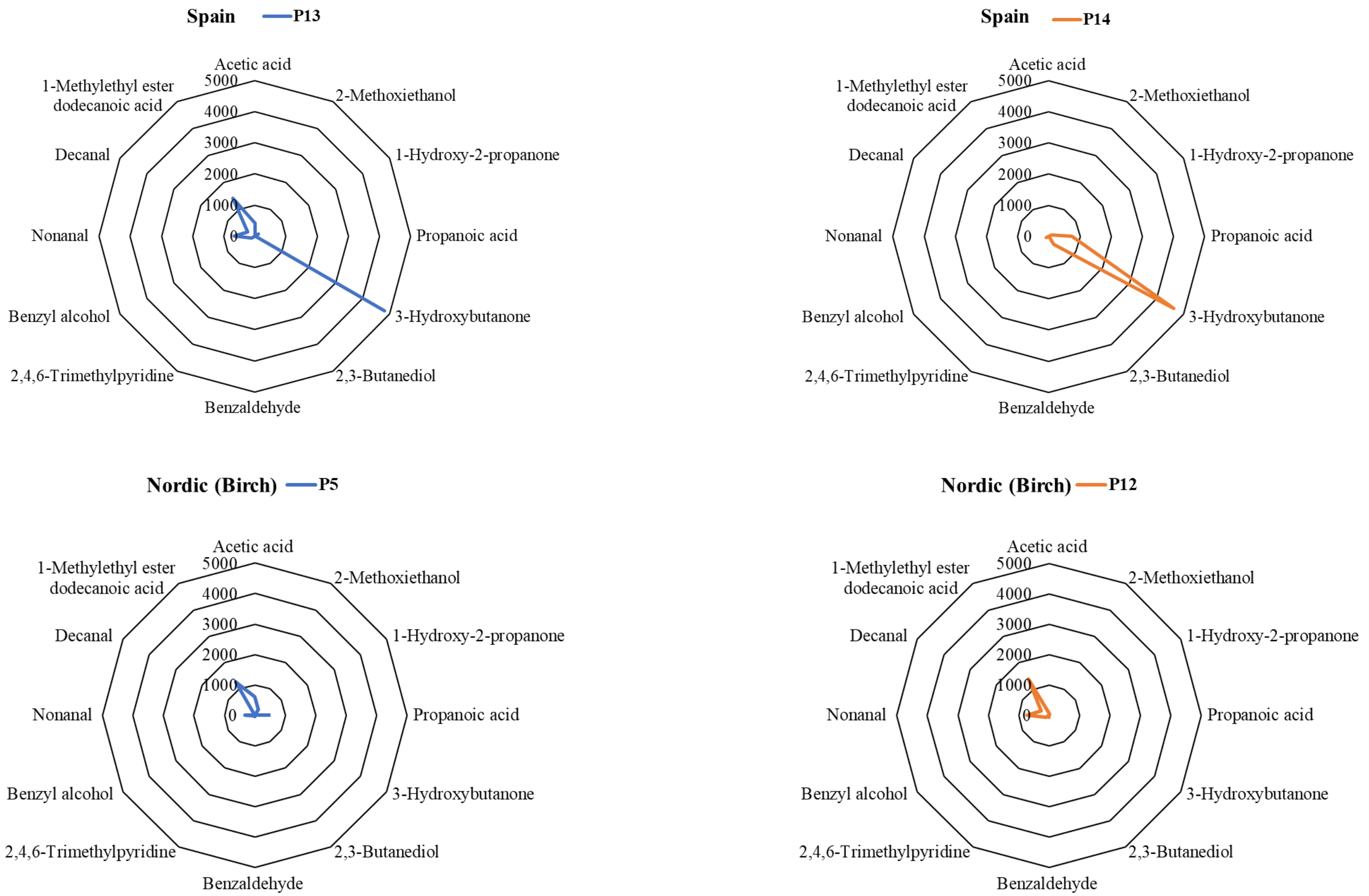
Spain
P13
Acetic acid
2-Methoxiethanol
1-Hydroxy-2-propanone
Propanoic acid
3-Hydroxybutanone
2,3-Butanediol
Benzaldehyde
2,4,6-Trimethylpyridine
Benzyl alcohol
Nonanal
Decanal
1-Methylethyl ester dodecanoic acid
5000
4000
3000
2000
1000
0
Spain
P14
Acetic acid
2-Methoxiethanol
1-Hydroxy-2-propanone
Propanoic acid
3-Hydroxybutanone
2,3-Butanediol
Benzaldehyde
2,4,6-Trimethylpyridine
Benzyl alcohol
Nonanal
Decanal
1-Methylethyl ester dodecanoic acid
5000
4000
3000
2000
1000
0
Nordic (Birch)
P5
Acetic acid
2-Methoxiethanol
1-Hydroxy-2-propanone
Propanoic acid
3-Hydroxybutanone
2,3-Butanediol
Benzaldehyde
2,4,6-Trimethylpyridine
Benzyl alcohol
Nonanal
Decanal
1-Methylethyl ester dodecanoic acid
5000
4000
3000
2000
1000
0
Nordic (Birch)
P12
Acetic acid
2-Methoxiethanol
1-Hydroxy-2-propanone
Propanoic acid
3-Hydroxybutanone
2,3-Butanediol
Benzaldehyde
2,4,6-Trimethylpyridine
Benzyl alcohol
Nonanal
Decanal
1-Methylethyl ester dodecanoic acid
5000
4000
3000
2000
1000
0

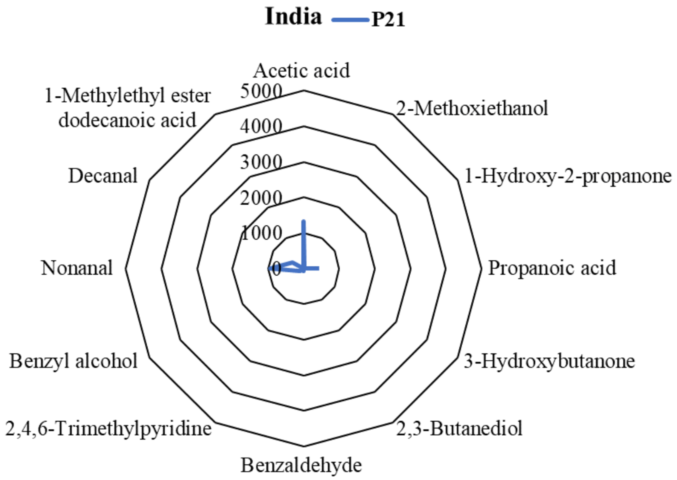

Figure S4 – Profile of volatile compounds obtained by SPME CAR-PDMS/GC-TOF-MS for each tested paper sample.
